# A bulk sub-femtoliter *in vitro* compartmentalization system using super-fine electrosprays

**DOI:** 10.1038/srep26257

**Published:** 2016-05-20

**Authors:** Bineet Sharma, Yuzuru Takamura, Tatsuya Shimoda, Manish Biyani

**Affiliations:** 1 Department of Bioscience and Biotechnology, Japan Advanced Institute of Science and Technology, 1-1 Asahidai, Nomi, Ishikawa 923-1292, Japan; 2 Center for Single Nanoscale Innovative Devices, Japan Advanced Institute of Science and Technology, 1-1 Asahidai, Nomi, Ishikawa 923-1292, Japan; 3 Biyani BioSolutions Pvt. Ltd., Biyani Research Group, R-4, Sector 3, Vidhyadhar Nagar, Jaipur 302023, India

## Abstract

The extreme miniaturization of biological and chemical assays in aqueous-droplet compartments enables spatiotemporal control for large-scale parallel experimentation and can thus permit new capabilities for “digitizing” directed molecular evolution methodologies. We report a remarkably facile bulk method to generate mega-scale monodisperse sub-femtoliter aqueous droplets by electrospray, using a prototype head with super-fine inkjet technology. Moreover, the electrostatic inkjet nozzle that injects the aqueous phase when immersed within an immiscible phase (an optimized oil/surfactant mixture) has the advantage of generating cell-like sub-femtoliter compartments for biomolecule encapsulation and successive biological and chemical reactions. Sub-femtoliter droplets of both liquid (water-in-oil, volumes ranging from 0.2 to 6.4 fL) and gel bead (agarose-in-oil, volume ranging from 0.3 to 15.6 fL) compartments with average sizes of 1.3 μm and 1.5 μm, respectively, were successfully generated using an inkjet nozzle at a speed of more than 10^5^ droplets per second. We demonstrated the applicability of this system by synthesizing fluorescent proteins using a cell-free expression system inside electrosprayed sub-femtoliter droplets at an accelerated rate, thereby extending the utility of *in vitro* compartmentalization with improved analytical performance for a top-down artificial cellular system.

With advances in micro/nano-scale devices, the encapsulation of biochemical reactions in extremely miniaturized aqueous-droplet compartments has redefined experimental design in the chemical and biological sciences^1^,^2^,^3^,^4^. A key role of such encapsulation based on water-in-oil microdroplet compartments, termed *in vitro* compartmentalization (IVC)^5^,^6^,^7^, has been to transform the use of natural selection in a test tube without needing to establish an absolute link between each protein (phenotype) and its encoding gene (genotype); such linkage has been a principal requirement of conventional molecular evolution but is often a limiting step. This ability of IVC highlights the fact that it can constitute a system that provides a route towards the conceptually simple and ‘bias-free’ *in vitro* directed evolution of functional biomolecules^8^; thus, the key issue examined in this report is the method of forming extremely miniaturized, monodispersed and controlled microdroplets in bulk for an *in vitro* evolutionary system.

The state of the art in IVC was originally developed based on the bulk emulsification of water-in-oil droplets using vortexing and homogenization. These methods are simple and fast, but yields are unpredictable and show considerable variability in droplet size, with typical diameters ranging from 1 to 100 μm, which correspond to fL-to-nL differences in reaction volume^9^,^10^. This polydispersity can lead to a potentially uneven distribution of evolving molecules that are typically very great in number (i.e., millions to quadrillions) across the population of droplets, thereby making precise and quantitative experiments difficult. In addition, the vigorous mixing that occurs during homogenization tends to result in an unacceptable loss of activity for the enzymes used in IVC^10^. Polydispersity limitations can be overcome by producing essentially monodisperse droplets in microchannels with a well-designed microfluidic geometry^11^. However, a microfluidic-based system has 2 substantial constraints. One constraint is a low production rate; i.e., the droplets are produced one at a time; the technique is also limited with respect to generating very small droplets. Attempts at overcoming the first constraint include the use of several successive microfluidic modules or advanced microchannel geometry that allow the serial formation of droplets at kilo-to-megahertz frequencies^12^,^13^,^14^. However, these methods are unable to incorporate the large libraries of evolving molecules that are used in evolutionary molecular engineering, as such libraries are several orders of magnitude larger than the upper limit of the microfluidic droplet numbers. In a different approach, microfabricated arrays of pico/femtoliter chambers using quartz or poly(dimethylsiloxane) (PDMS) have also been developed but again are technically limited to low throughput (on a kilo-to-mega scale)^15^,^16^,^17^,^18^. Pivotally, the production rate of an *in vitro* gene expression system was reported to be inversely proportional to the radius of the droplets^4^,^19^ and was also strongly affected by the nature of the oil/surfactant used for droplet generation in bulk emulsion^20^,^21^. Therefore, a new strategy that can integrate the principal features of conventional emulsification, i.e., bulk formation and microfluidic approaches, and monodisperse droplet formation, is an exciting prospect that may allow the ultrahigh-throughput generation of highly monodisperse and extremely miniaturized droplets (sub-femtoliter) that can maintain a sizeable population of evolving molecules in IVC-based directed evolution.

Electrospray, an ionization technique typically used in mass spectrometry, can be a potential means to create bulk monodisperse droplets^22^. The electrospray is caused by an electric force, which acts on the surface of a liquid at a capillary nozzle outlet to form the Taylor cone; when the electric field force exceeds the surface tension, a jet elongates into a fine filament that then spontaneously breaks up into fine and relatively monodisperse droplets that are much smaller than the orifice diameter. This technique has been widely recognized, including by a Nobel Prize in chemistry; however, its use in an immersed mode is still limited, mainly because of a lack of knowledge about the phenomenon when the dispersing fluid is immersed in another immiscible liquid^23^. The electro-dispersion of a liquid inside an oil could be a technique of interest to fulfill the aforementioned objectives. Interestingly, the electro-dispersion of a liquid in a bath of another liquid has been reported to be much easier than in air^24^,^25^.

In this study, we developed a simple new platform that combines immersed electrospray with IVC by using super-fine inkjet technology (SIJ Technology, Inc.) for the ultra-rapid generation of water-in-oil droplets. Our platform enables improved size and speed control compared to conventional approaches (bulk or microfluidic), and it permits the massive production of parallel IVC. The electrostatic-based inkjet nozzle is submerged in the host oil phase; when the voltage is applied, this system is able to continuously produce biomolecules that are encapsulated in sub-femtoliter droplets. We systematically investigated the parameters that affect droplet size and monodispersity, including electrical parameters such as voltage and frequency, orifice diameter size, and fluid properties. To demonstrate the applicability of our approach, we produced electro-dispersed water-in-oil droplets that encapsulated cell-free gene expression systems producing green fluorescent protein (GFP) and/or mCherry. Additionally, we monitored the rate of protein expression as a function of droplet size by examining fluorescence intensity using confocal fluorescence microscopy. To the best of our knowledge, this is the first report describing the application of immersed electrosprays for sub-femtoliter IVC studies. The methodologies developed here are straightforward to set up and should be useful for quantifying the yield of active proteins and for controlling gene expression noise in *in vitro* droplets for directed molecular evolution studies.

## Results and Discussion

### Producing super-fine electrospray-based sub-femtoliter IVC

**Figure 1:**
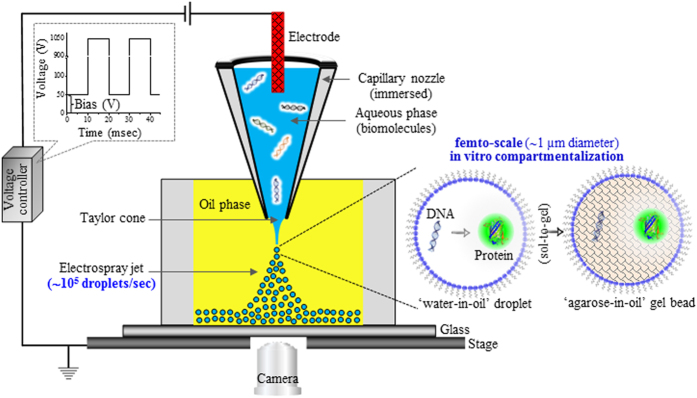
Immersed super-fine electrospray-based miniaturized *in vitro* compartmentalization. A schematic illustration of the experimental setup is shown. An inkjet nozzle head (filled with a nanomolar solution of template DNA and a cell-free gene expression system) immersed in an immiscible oil phase can ultra-rapidly produce monodisperse water-in-oil femtoliter-scale droplets in bulk on a mega-scale. The enlarged diagram shows the encapsulation of the *in vitro* protein expression system in a water-in-oil droplet or in an agarose-in-oil gel bead.

**Figure 2:**
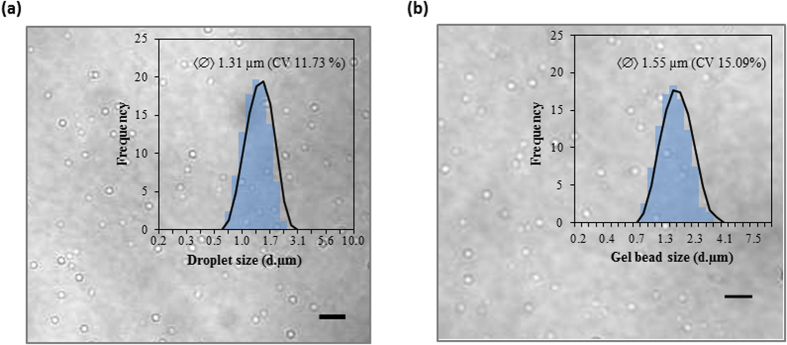
Size measurement for femto-scale *in vitro* compartmentalization. Confocal microscopic image of water-in-oil droplets (**a**) and agarose-in-oil gel beads (**b**). The droplet size distribution was measured by dynamic light scattering. Inset: Histogram of the droplet size distribution obtained using a 4-μm (**a**) and 15-μm (**b**) nozzle orifice diameter. The mean diameter is 1.31 μm, with a volume of 1.2 fL (**a**) and 1.55 μm, with a volume of 1.9 fL (**b**). The coefficients of variation (CV) is 11.73% (**a**) and 15.09% (**b**). Scale bar: 5 μm (**a**) and 10 μm (**b**).

We report here a technique that uses super-fine inkjet technology (SIJ Technology, Inc.) as an electrospray mode to generate monodisperse-droplet jets with diameters in the sub-micrometer range. The principle is similar to electrospray techniques in which a conducting liquid is slowly injected through an electrified capillary nozzle. The liquid inside the capillary nozzle attains a hemispherical meniscus because of its surface tension. With an applied pulsed DC voltage, a tangential electric stress appears as a result of the accumulation of ions near the meniscus area. At a particular threshold voltage, a Rayleigh instability phenomenon occurs, causing an imbalance between the electrostatic force and the surface tension acting on the surface of the water. This results in the formation of a conical shape, commonly termed the Taylor cone, and a thin microthread of liquid issues from the tip of the Taylor cone, which eventually fragments to form a spray of monodisperse droplets. Importantly, the size of the generated droplets is independent of the diameter of the capillary tube, and droplets smaller than a micrometer can be obtained. The electrospray of water-in-oil droplets for IVC can be generated by immersing the nozzle in an immiscible phase, i.e., oil. The basic experimental setup for the technique is depicted in Fig. 1. A capillary nozzle that is connected to a high-voltage supply controller unit through a fine electrode wire is immersed in a clear-bottomed reservoir (for visualization) filled with a mixture of oil/surfactant. The electrical field is generated by applying a high-voltage drop from the capillary nozzle, and a liquid consisting of biomolecules is extruded and sprayed through a nozzle. As schematically illustrated in Fig. 1, water-in-oil femto-scale compartments are formed by the generation of monodisperse aqueous droplets followed by their mutual diffusion into a mixture of oil/surfactant. Real-time (see Supplementary Fig. S1 and Supplementary Movie S1) and high-speed (see Supplementary Fig. S2 and Supplementary Movie 2) imaging was used to capture the release of a jet breaking into discrete droplets. The corresponding video can be seen in the supporting movies. Laser scanning confocal microscopy was used to image the water-in-oil droplet compartments. As shown in Fig. 2a, the average size of the droplets was calculated by dynamic light scattering and was found to be 1.31 μm in size when using a nozzle with a 4-μm orifice diameter. To calculate of the number of droplets generated by an electrospray system, 2 types of high-speed video cameras were used. A camera at 10,000 frames per second (fps) (Phantom VR 502, Vision Research) was used to capture the droplets generated in one pulse; the number of generated droplets was then calculated using ImageJ software (see Supplementary Fig. S2). A second camera was operated at 16,000 fps (K5, Kato Koken) with MotionV, a fully automatic 2-dimensional motion analysis program (see Supplementary Movie S3). According to our calculations, 55 to 108 droplets were produced in a single pulse using the experimental setup (Fig. 1). At the maximum frequency of 1 kHz, i.e., 10^3^ pulses per second, the current setup leads to the generation of monodisperse aqueous droplets at rates near 10^5^ droplets per second. The production rate can be further increased by approximately 2 orders of magnitude by using higher-frequency (e.g., 20 kHz in our another experimental setup) electrostatic waves. Further advantages and perspectives of electrospray-based droplet generation system over other available droplet generation systems can be found as Supplementary Table S1.

Furthermore, to perform directed molecular evolution inside these tiny femtoliter droplets, it is desirable to exchange the solution or to add multiple washing steps for the removal of undesired molecules. However, such steps can be problematic when using a freestanding oil layer with water-in-oil droplets. Recently, the application of a hydrogel matrix, such as agarose, inside the droplet has been greatly anticipated as a matrix support for multistep processes, as small molecules can be exchanged while the gel matrix retains larger and more desirable molecules such as proteins. Moreover, the effect of the support (hydrogel) matrix on enzyme activity can be more pronounced in comparison with an unconfined enzyme in water^26^. When employing this strategy, the use of low-melting-temperature agarose confers a flexible sol-gel switching property. Therefore, next we evaluated the ability of our system to produce monodisperse femtoliter hydrogel-in-oil gel beads, which are difficult to obtain by conventional microfluidic processes. We encapsulated ultra-low-gelling agarose, which has a melting point of approximately 55 °C and a gelling point below 15 °C, with fluorescent dye inside water-in-oil droplets. As shown in Fig. 2b, the average size of the agarose-in-oil gel beads was calculated by dynamic light scattering and was found to be 1.55 μm in size when using a nozzle with a 15-μm orifice diameter. Following electrospray, the generated agarose-in-oil droplets were converted into agarose-in-oil gel beads upon cooling (from 37 °C to 4 °C). For imaging, the oil boundary on an agarose hydrogel bead are removed by breaking the emulsion twice in the presence of acetone and analyzed by transmission electron microscopy (TEM) (see Supplementary Fig. S3). The average size of the beads after removing the oil boundary was ~1.75 μm, corresponding to a volume of ~2.8 fL.

### Optimizing the parameters for monodisperse femtodroplet generation

**Figure 3:**
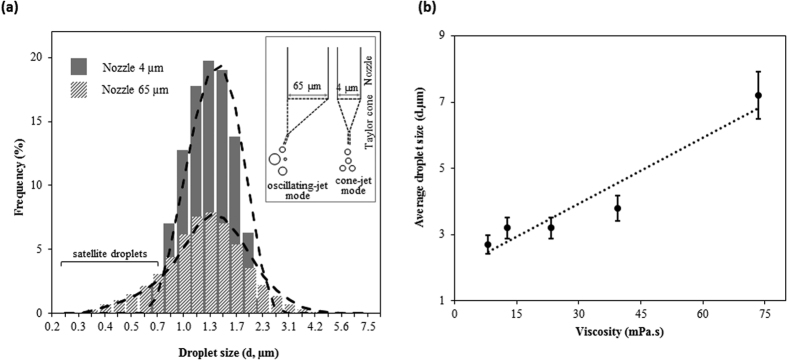
Influence of electrospray parameters on water-in-oil droplet size distribution. (**a**) Comparison of nozzles with bigger (∼65 μm) and smaller (~4 μm) orifice diameters. Inset: The bigger nozzle exhibits an oscillating jet mode, which results in a polydisperse distribution with satellite droplets; the small nozzle produces highly monodisperse droplets because of its cone-jet mode. (**b**) Comparison of droplet sizes in oil/surfactant mixtures with different viscosities, prepared by changing the proportion of Tegosoft DEC while keeping the ABIL EM 90 and mineral oil at a fixed ratio. All observations were performed at a bias voltage of 50 V and a frequency of 100 Hz.

In a typical inkjet, parameters such as viscosity and surface tension are key factors that affect droplet size. Because we used an electrostatic inkjet with a dipped nozzle as an electrospray method, the disintegration of the jet into different droplet sizes is predominantly affected by the size of the nozzle, the surface tension of the aqueous phase, the viscosity of the oil phase, and various instrumental parameters, including the voltage supply, bias and frequency. Therefore, the optimal conditions of these parameters for generating monodisperse and stable droplets were determined. First, the size of the nozzle determines the geometry of Taylor’s cone, and different modes (e.g., cone-jet or oscillating-jet) can be generated in the jet spray, depending on the orifice diameter of the nozzle. Therefore, the size of the nozzle plays an important role in the jet spray mode when optimizing the position and precisely controlling the volume of the droplets. Figure 3a shows the droplet size distribution for 2 different nozzle sizes, 4 μm and 65 μm, while keeping other parameters constant. A large nozzle provides a large interface area; thus, under the influence of an external electric field, each liquid drop experiences imbalance stresses at the interface, resulting in an oscillating jet mode that leads to a wider droplet size distribution. In contrast, a small nozzle provides less interface area, leading to a cone jet-mode. Hence, polydispersity is observed in the case of a bigger nozzle compared to a smaller one. Second, the area of the plume is determined by the medium in which the spray occurs. Because air provides a negligible friction force, droplet generation in air by inkjet printing is more convenient than in the case of oil, where a viscous force (or friction force) applies a shear stress at the interface. However, compared to air, an oil medium increases the stability of water droplets, with fewer possibilities of generating satellite droplets. Hence, we investigated the effect of the oil-phase viscosity on the average droplet size. Oil phases with different viscosities were prepared by mixing mineral oil, emollient (Tegosoft DEC, an oil with low viscosity), and surfactant (ABIL EM 90, a nonionic emulsifier with high viscosity) at different ratios (see Supplementary Table S2). We observed that the average droplet size increased with increasing viscosity, as shown in Fig. 3b and Supplementary Fig. S4. In addition, the stability of generated droplets was evaluated against elevated high-temperature (90 °C for 5 min) and storage time (25 °C for 24 h). No significant effect on the average sizes of droplets was observed (see Supplementary Fig. S5). Third, it is easy to speculate about the dependency of voltage and frequency on the droplet size distribution, as the electrospray occurs when a voltage is applied to the liquid, and the frequency of the applied voltage can affect the forces acting at the interfaces. Similar to the flow rate factor in an electrohydrodynamic inkjet^27^,^28^, voltage plays the same role in an electrostatic inkjet. An increase in the applied bias voltage increases the amount of liquid at the interface, yielding larger droplet sizes. Consistent with this relationship, we observed a shift towards a larger droplet size when increasing the bias voltage from 100 V to 1000 V at a constant frequency of 100 Hz (see Supplementary Fig. S6 (a). However, a higher frequency at a higher voltage encourages a smaller droplet size because of increased disturbance at the interface of the aqueous and oil phases, which results in smaller droplets compared to a lower frequency (see Supplementary Fig. S6 (b). Following our observations, optimal parameters for producing small, monodisperse droplets were set as follows: a nozzle with a 4-μm orifice diameter, a less-viscous (near 8 mPa.s) oil/surfactant mixture and electrostatic conditions using a higher voltage (1,000 V_bias_) and frequency (1,000 Hz). A stable droplet generation were achieved using a minimum 2.6% nonionic surfactant (Cetyl PEG/PPG-10/1 dimethicone, Abil EM 90) which provides both a stable droplet emulsion against flocculation and coalescence and sufficient compatibility with the following reaction against high temperature or incubation time.

### Quantification of *in vitro* protein expression in sub-femtoliter droplets

**Figure 4:**
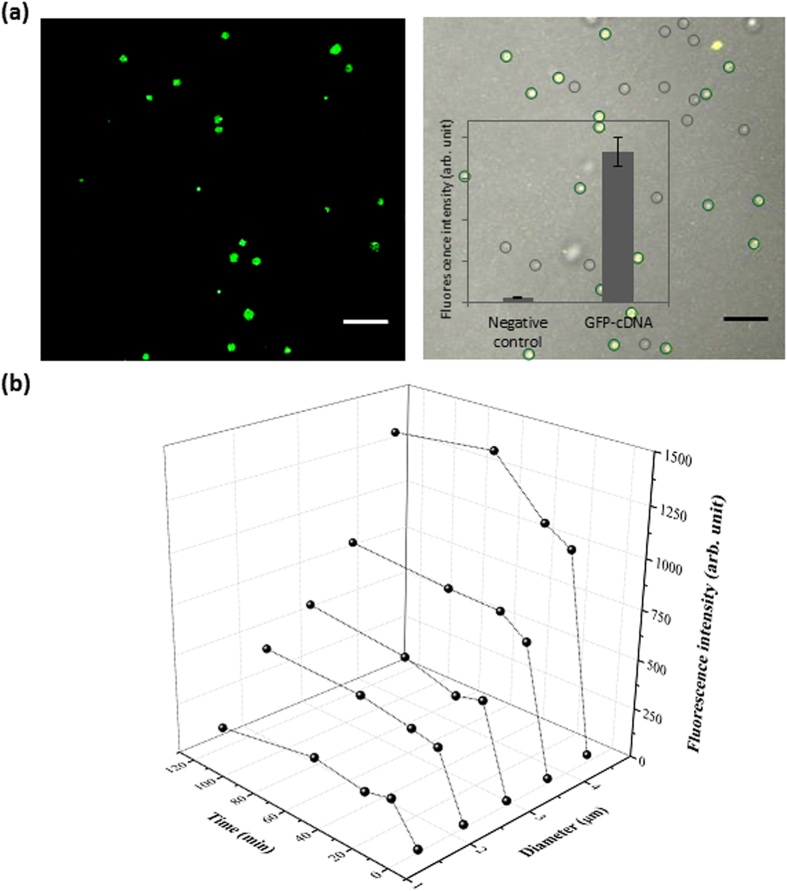
*In vitro* protein expression in sub-femtoliter water-in-oil droplets generated by electrospray. (**a**) The figure shows a confocal fluorescence image alone (left) or merged with the corresponding brightfield image for GFP synthesized in femtoliter water-in-oil droplets. Green and grey encircled droplets represent GFP-filled and empty droplets, respectively. The graph shows the fluorescence intensity of droplets with and without (negative control) the template DNA for GFP synthesis. Scale bar: 15 μm. (**b**) Time courses for the synthesis of GFP in 5 differently sized droplets (1.8 fL, 5.5 fL, 13 fL, 25.5 fL, and 44.3 fL). The fluorescence of GFP reached a plateau at an earlier time (<15 min) in smaller droplets. The results represent the average data from 176 different droplets.

As a first step in demonstrating the utility of super-fine electrosprays for IVC purposes in which genes are compartmentalized and expressed, GFP synthesis inside femtoliter droplets was performed by encapsulating a GFP-encoding gene with a PURE system as a cell-free gene expression system in femtoliter droplets. GFP-encoding cDNA (35 nM) with a PURE expression system was placed in a 4-μm glass nozzle, and water-in-oil droplets were generated using electrosprays before being incubated for 2 h at 37 °C. As shown in Fig. 4a, GFP fluorescence was successfully monitored using confocal microscopy. Because a negligible amount of fluorescence was detected in droplets generated in the absence of GFP-cDNA, it seemed obvious that the fluorescence was due to the accumulation of newly synthesized GFP inside the droplets. The fluorescence intensity graph in Fig. 4a clearly depicts the difference between the control (without template) and the sample (with template). The smallest droplet in which we observed GFP fluorescence was 0.81 μm in diameter, corresponding to a volume of 0.3 fL.

To further evaluate whether the applied high voltage influences the biological activity or behavior of biomolecules, we electrosprayed droplets with pre-synthesized GFP (bulk synthesis; so-called ‘off-droplet’ synthesis) and compared the fluorescence intensity of these droplets with droplets that were electrosprayed using the GFP-encoding gene and *in vitro* gene expression system (so-called ‘on-droplet’ synthesis). The GFP fluorescence increased with the size of the droplets and was nearly identical in smaller volumes for ‘on-droplet’ and ‘off-droplet’ synthesis (see Supplementary Fig. S7), suggesting that bio-electrospraying neither affects protein function nor the ability of an *in vitro* gene expression system to synthesize functional proteins; thus, it is reasonable to neglect any adverse influence of high-voltage electrosprays on the efficiency of protein expression for IVC in the designed system. Moreover, the production in ‘on-droplet’ GFP synthesis in larger volumes was comparatively higher than that in ‘off-droplet’, a result that is similar to a previously reported observation^19^ showing that confinement of PURE system components in microdroplets can accelerate the turnover reaction of protein synthesis. In our earlier work using a microreactor array chip, we observed that the synthesis of GFP inside a microchamber with a diameter and depth of 4 μm, corresponding to a volume of 50 fL, reached a plateau after approximately 90 min of synthesis, which is identical to that of bulk synthesis at the μl-scale volume. Very interestingly, the maturation level was observed to reach a plateau earlier when synthesis occurred inside a smaller, fL-scale droplet (<50 fL). A time-course study on GFP expression in differently sized droplets, from 1.5 μm to 4.4 μm in diameter (corresponding to volumes of 1.8 fL to 44.3 fL), is presented in Fig. 4b. The increase in GFP fluorescence stopped earlier (within 15 min of the start of protein expression) in smaller volumes, while it stopped after 30 min in larger volumes. However, the fluorescence intensity peak gradually increased with increased droplet volume. This result confirms that protein synthesis is accelerated in smaller droplets and that the peak of the plateau is the result of available PURE system components (e.g., nutrient and energy molecules) for further turnover reactions, which is clearly less in smaller droplets because a smaller volume has fewer components. As a result, this system can be used to access quantitative information on the rate-determining step of cell-free protein synthesis in femtoliter-scale droplets.

We then attempted to evaluate the expression of GFP in femtoliter gel beads produced by our system. A gel matrix retards the diffusional migration of components of the cell-free system (approximately 80 different macromolecular species in the PURE system^29^) and can thereby accelerate the reaction because of improved gene stability, higher local component concentration and a faster enzyme turnover rate. A hydrogel system has been reported to improve the efficiency of protein synthesis by approximately 300 times in comparison with a solution-based system^30^. As expected, we also observed higher GFP fluorescence in agarose-in-oil gel beads than in similarly sized water-in-oil droplets (see Supplementary Fig. S8). This result confirms that the described electrospray system will find many applications that employ a gel matrix. Very recently, the synthesis of GFP inside emulsion droplets was reported to depend on the droplet interface structure^19^,^20^. The type of oil/surfactant mixture was observed to have a significant effect on the amount of active protein expression inside the droplets; thus, such an interfacial phenomenon at the membrane surface of each droplet can be manifested by minimizing the droplet size using our system, as the surface area-to-volume (S/V) ratio increases with decreasing droplet size. Therefore, cell-free expression in the droplets generated by our system not only can enable the control of reactions in sub-femtoliter volumes but also can permit studying the dynamic effects of volume on protein synthesis.

### The dynamics of ‘solute entrapment’ in femtoliter droplets

Our results show that an electrostatic inkjet system can provide a facile platform to efficiently produce bulk quantities of monodisperse water-in-oil or gel-in-oil droplets that can be very powerful for applications that rely on highly miniaturized and controlled *in vitro* compartmentalization. After characterizing our system, we quantified the cell-free protein synthesis dynamics in the extremely miniaturized droplets that are produced by this system at sub-femtoliter volumes. A template DNA encoding GFP was extremely diluted, from nM-to-fM, to evaluate the effect of template concentration on the rate of protein synthesis. A time-course study was performed in droplets sized near 5 μm (corresponding to a volume of 65 fL) and near 1 μm (corresponding to a volume of 2 fL) at concentrations of 35.75 nM and 35.75 fM (see Supplementary Fig. S9). We estimated that approximately 1,300 copies of the template DNA are encapsulated in each droplet at a 35.75-nM concentration. Hence, further extreme dilution by 6 orders of magnitude would encapsulate approximately one DNA molecule in one droplet per 10^3^ droplets, thus leading to an approximately 1000-fold drop in fluorescence. Interestingly, this expected result was not observed, as both tested concentrations showed an identical fluorescence of GFP synthesis during the initial 15 min of the reaction, respective to their droplet sizes (5 μm or 1 μm) (Supplementary Fig. S9). This suggests that the reaction rate of GFP synthesis in the initial 15 min is significant and fast enough, even in the case of lower template concentration, to hit the first plateau-level determined by the droplet sizes (higher in bigger and lower in smaller droplets) and then turned to slow for subsequent time course of reaction. These results are supportive to our earlier observation on the effect of the amount of DNA template on the production of GFP inside a microchamber array with a diameter range between 20 and 100 μm^31^, where no significant difference was observed in smaller microchambers. This phenomenon is interesting and may suggest possibilities of two reaction paths: i) fast and significant in smaller droplets for both the concentrations, and ii) slow and less-significant in larger droplets for only higher template concentration. We are considering that the former reaction path is enhanced and controlled by the surface of the droplet^19^, and the later reaction path is mainly governed by the rest of the reactions in the bulk solution of the droplets.

To further evaluate this, we co-expressed 2 recombinant genes inside the femtodroplets using 2 different but extremely diluted concentrations (nM to fM). For this purpose, we used 2 fluorescent proteins, GFP (green) and mCherry (red), that are commonly used to enable dual protein labelling and can be co-expressed in a cell-free system. An equal mixture of GFP-cDNA and mCherry-cDNA at a concentration of 17.87 nM or 17.87 fM was mixed with the PURE system, and the resulting mixture was encapsulated in water-in-oil droplets by electrospray. A nanomolar-level concentration would make probable the encapsulation of both cDNAs in each single droplet, whereas a femtomolar concentration would yield 1 copy of cDNA in 1 out of 10^3^ droplets. However, again we did not observe a significant difference in the results for either of the 2 different concentrations, except that more empty droplets (with no fluorescence) were seen at the femtomolar concentration (see Supplementary Fig. S10). In comparison to green fluorescence, a smaller number of red fluorescent droplets were observed, which might be due to the very fast chromophore maturation time (almost 10-fold faster) of GFP relative to mCherry^32^. These observations support the hypothesis that the encapsulation of biomolecules in extremely diluted conditions, such as at a DNA concentration of 17.87 fM, tends to exhibit the ‘solute entrapment’ or ‘super-concentration’ effect^33^ characterized by many empty droplets and very few droplets (less than 8%) with either green/red or yellow (both) fluorescence. Noteworthily, the ratio of empty (negative) and positive droplets was 1:1.9 when a higher concentration (nanomolar-scale) of template were used (see Supplementary Table S3). This phenomenon suggests that the extremely miniaturized femtoliter compartments generated by our system can be useful to induce a ‘super-concentration’ effect and thus can lead to the production of a remarkable rate-acceleration effect in a complex biochemical reaction in a sub-femtoliter IVC-based artificial cellular system.

## Conclusion

In summary, this paper describes a facile electrospray-based approach for the bulk production of robust artificial cell-like compartments on a minimal biochemical reaction scale for maximum reaction output and accelerated reaction rates. We show that engineered sub-femtoliter-scale aqueous droplets or gel bead compartments made by the application of electrospray technology are expected to facilitate not only sensitivity improvements in top-down artificial cellular systems but also a colossal leap in directed molecular evolution methodologies.

## Methods

### Electrospray setup and procedure

The water-in-oil droplet generation system was created by using a prototype head with super-fine inkjet technology [SIJ Technology, Japan]. A small hole with an approximately 6-mm diameter was punched into commercially available silicone rubber (3 cm × 3 cm × 3 mm) and placed on a glass slide (35 × 55 mm) to construct a chamber for droplet collection (Fig. 1). Laboratory-made glass nozzles (~65 μm orifice diameter) and commercially available glass nozzles (~4 μm orifice diameter) [SIJ Technology, Japan] were used throughout the experiments. A 90-mm glass capillary was used to make a ~65 μm nozzle with a Puller machine PC-10 [Narishige, Japan] using a 2-step mode and keeping the heater level between 60 and 50. Tungsten wire was used as an electrode when the large nozzle was used. An oil phase containing 50% ABIL EM 90 [Evonik Industries], 36% Tegosoft DEC [Evonik Industries], and 14% mineral oil [Sigma Aldrich] was used. Oil mixtures with different viscosities were prepared by changing the proportion of Tegosoft DEC from 10% to 90% while keeping the ABIL EM90 and mineral oil at a ratio of 2.85:1. Oil viscosities were measured with a Viscomate [VM-10A, Sekonic CBC Co. Ltd]. All of the oil mixtures used in the experiments were freshly prepared by vortexing at 2,500 rpm for 5 min and incubating at 30 °C for 30 min.

### Water-in-oil droplet generation

The ~4-μm glass nozzle was filled with 7 μl of nuclease-free water and was then fixed to the inkjet machine. The oil phase (100 μl) was poured into the oil chamber, and the glass nozzle was immersed in oil. A voltage (V_max_ = 1000 V, V_bias_ = 50 V) was applied through the submerged glass nozzle containing the aqueous solution caused the solution to jet into the cavity with the oil phase, producing a large amount of water-in-oil droplets at a generation speed of ~10^5^ droplets per second. To calculate the droplet size and distribution by dynamic light scattering (DLS), a [Malvern Zetasizer Nano ZS] machine was used. The average droplet size was also measured using a laser scanning confocal microscope [Olympus FluoView 1000] with the help of ImageJ software. A high-speed camera [Phantom VR 502 or Kato Koken K5] was used to estimate the water-in-oil droplet generation speed.

### Transmission electron microscopy (TEM) observation

An agarose solution (1.0%) [Type IX-A, Ultra-low gelling temperature, Sigma-Aldrich] with or without a gene expression system was pipetted into a ~15-μm glass nozzle for the generation of agarose-in-oil gel beads. Upon voltage (500 V) application, agarose droplets in oil were generated by a dipped nozzle, and the oil chamber was then quickly transferred to 4 °C for 30 min to permit polymerization and conversion of the agarose droplet into a gel bead. For TEM observation, the sample was prepared by washing the gel beads twice in acetone to remove the oil boundary and centrifuging at 100 × g for 2 min. The washed gel beads were then dropped onto a copper grid and dried at room temperature overnight in a vacuum. TEM images were recorded with an H-7100 machine (Hitachi, Ltd.) operating at 100 kV.

### *In vitro* protein expression in droplets

The templates that were used for *in vitro* expression (coupled transcription/translation) were prepared by PCR amplification of plasmids encoding GFPuv4 and mCherry (Clontech, Takara Bio Inc., Japan) followed by purification using a QIAquick PCR purification kit (Qiagen, Japan). The DNA concentrations were determined by absorbance at 260 nm using a NanoDrop 2000 UV-Vis spectrophotometer. Two and a half microliters of purified cDNA template encoding green fluorescent protein (GFPuv4, the brightest variant of GFPuv) or mCherry was gently mixed with a commercial coupled transcription/translation system (PURExpress, NEB) containing solution A (5 μl) and solution B (3.75 μl). To avoid the loss of template DNA or other components of the cell-free system by adsorption onto the inner glass surface of the nozzle, the nozzle was pre-treated by rinsing it with an aliquot of the reaction mixture followed by pipetting fresh solution into the glass nozzle to generate droplets in oil using an electrostatic inkjet system. The water-in-oil droplets were generated in 100 μl of an oil/surfactant mixture at 50 V and 10 Hz. All of the droplets were transferred to an Eppendorf tube and were incubated at 37 °C for 2 h to permit cell-free expression. For microscopic studies, a confocal laser scanning microscope [Olympus FluoView 1000 spectral-based] was used to capture brightfield and fluorescence images of the droplets. A laser light with an Ar 488-nm wavelength was used to excite the GFP, which was observed through an Alexa Fluor 488 green dye filter. All images were captured using a 60× lens that was focused on the equatorial section. For the time-course study, the above procedure was performed using different aliquots that had been prepared for each time interval. The vials containing droplets with the cell-free expression system were quenched at a specific time and were observed with confocal microscopy.

## Additional Information

**How to cite this article**: Sharma, B. *et al*. A bulk sub-femtoliter *in vitro* compartmentalization system using super-fine electrosprays. *Sci. Rep.* **6**, 26257; doi: 10.1038/srep26257 (2016).

## Supplementary Material

Supplementary Information

Supplementary Movie S1

Supplementary Movie S2

Supplementary Movie S3
