# Supplementary material for: A bulk sub-femtoliter in vitro compartmentalization system using super-fine electrosprays
Source: Sci Rep. 2016 May 20;6:26257. doi: 10.1038/srep26257 (PMC4873800; doi:10.1038/srep26257)
Supplement: Supplementary Information [file srep26257-s1.pdf]

Supplementary Information

A bulk sub-femtoliter *in vitro* compartmentalization system using super-fine electrosprays

Bineet Sharma[1], Yuzuru Takamura[1], Tatsuya Shimoda[1], Manish Biyani[1,2,*]

[1]Department of Bioscience and Biotechnology, Japan Advanced Institute of Science and Technology, 1-1 Asahidai, Nomi, Ishikawa 923-1292, Japan

[2]Center for Single Nanoscale Innovative Devices, Japan Advanced Institute of Science and Technology, 1-1 Asahidai, Nomi, Ishikawa 923-1292, Japan

Supplementary Figure S1: Photograph of the experimental setup of the inkjet system for water-in-oil droplet generation showing the oil chamber on the glass slide lying on the stage. The potential difference between the tungsten electrode and the stage (which is grounded) caused dispersion of the aqueous phase in a continuous oil phase. The real-time movie shows water-in-oil droplet generation using a 4-µm nozzle size at 50 V and 100 Hz.

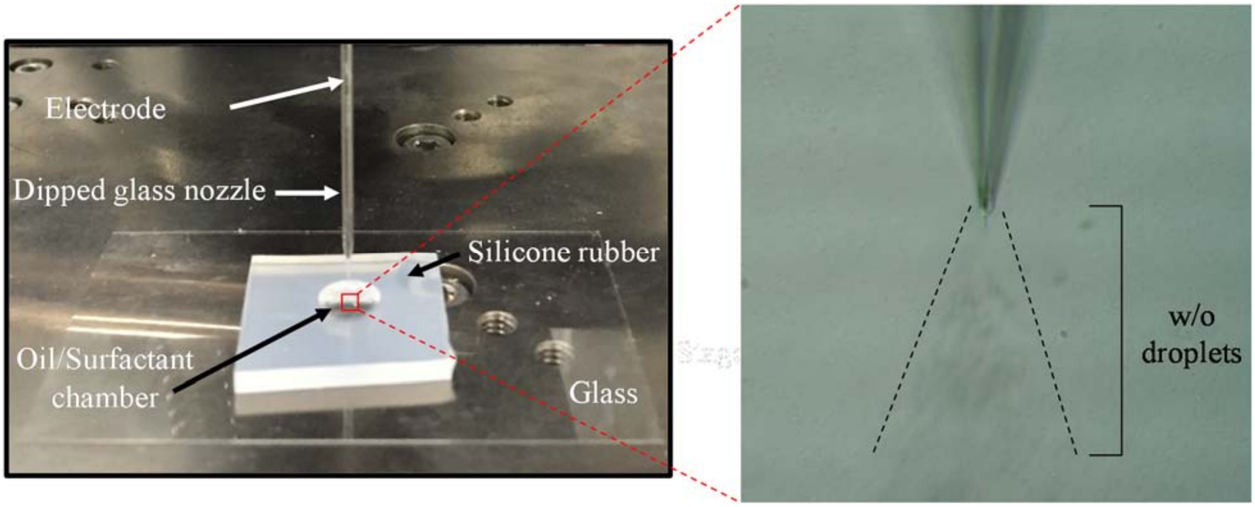

Supplementary Figure S2: Calculation of the number of droplets in image (a) using ImageJ software. Panels 1-7 show surface plots of the different regions, which show the number of droplets generated by one pulse using a high-speed camera (Phantom VR 502). More than 55 droplets were counted in 1 pulse at 50 V. Nozzle, ~65 μm.

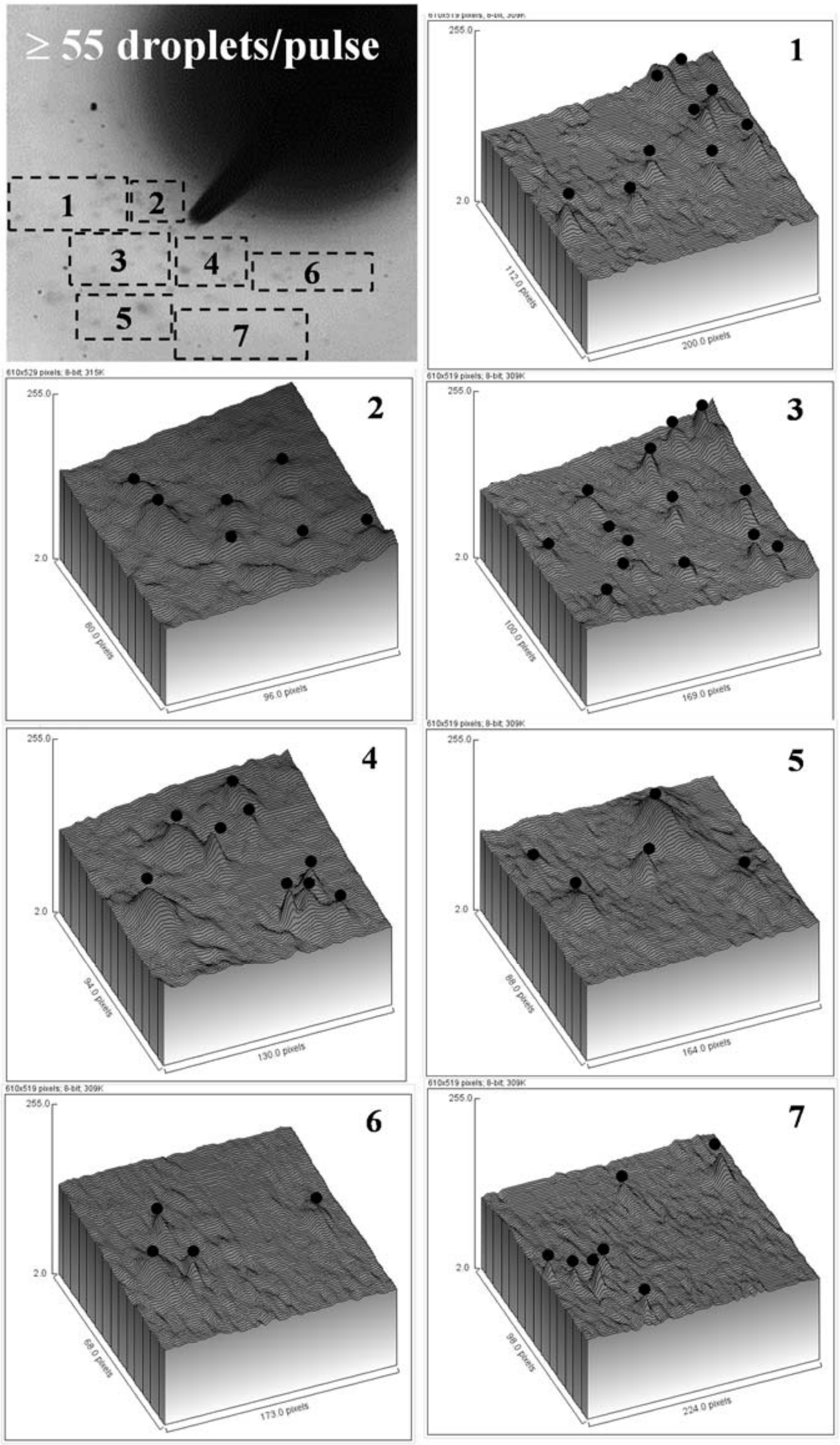

Supplementary Figure S3: TEM image of agarose-in-oil gel bead. Inset: Histogram of the droplet size distribution obtained using a 15-μm nozzle orifice diameter and calculated by ImageJ. The mean diameter is 1.75 μm, with a volume of 2.8 fL. The coefficients of variation (CV) is 17.13%. Scale bar: 1 μm.

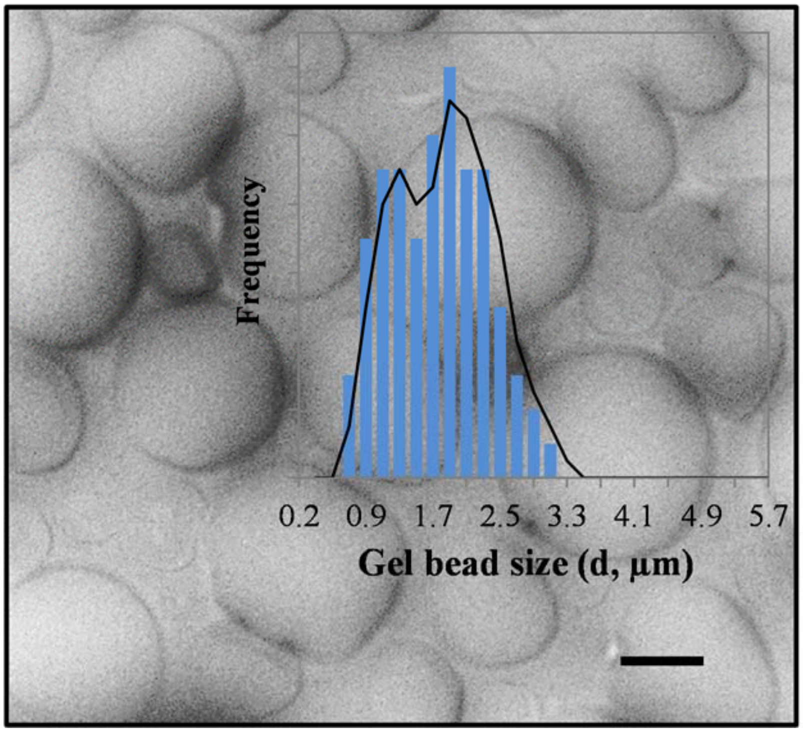

Supplementary Figure S4: Fluorescence image of water-in-oil droplets in oil phases with different viscosity. As the viscosity decreased from 73.4 to 8.06 mPa s, the droplet size also decreased, as shown in panels (a) – (e). Scale bars, 35 μm.

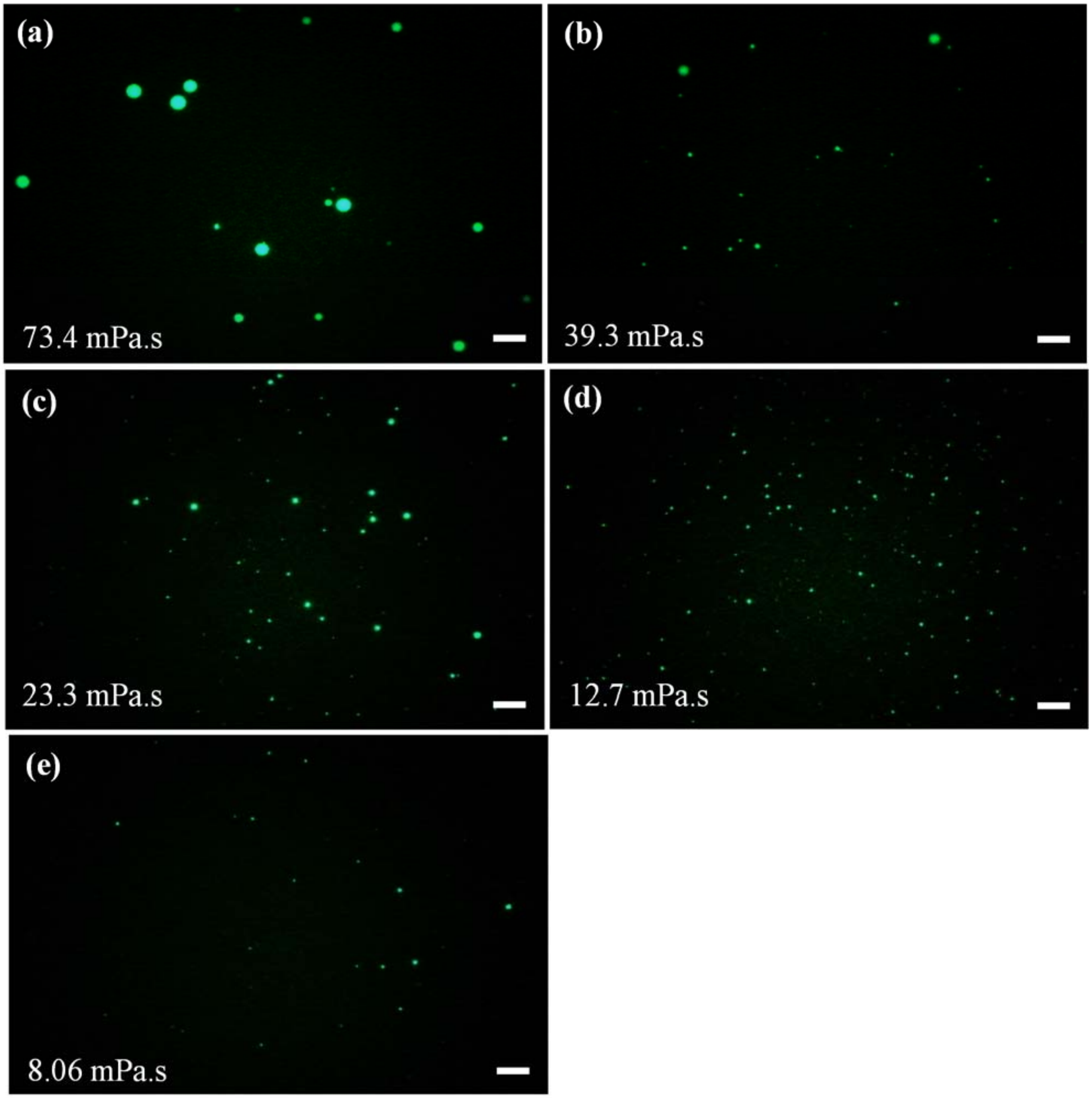

Supplementary Figure S5: Droplet size distributions before (a) and after (b) temperature treatment (90°C, 5 min).

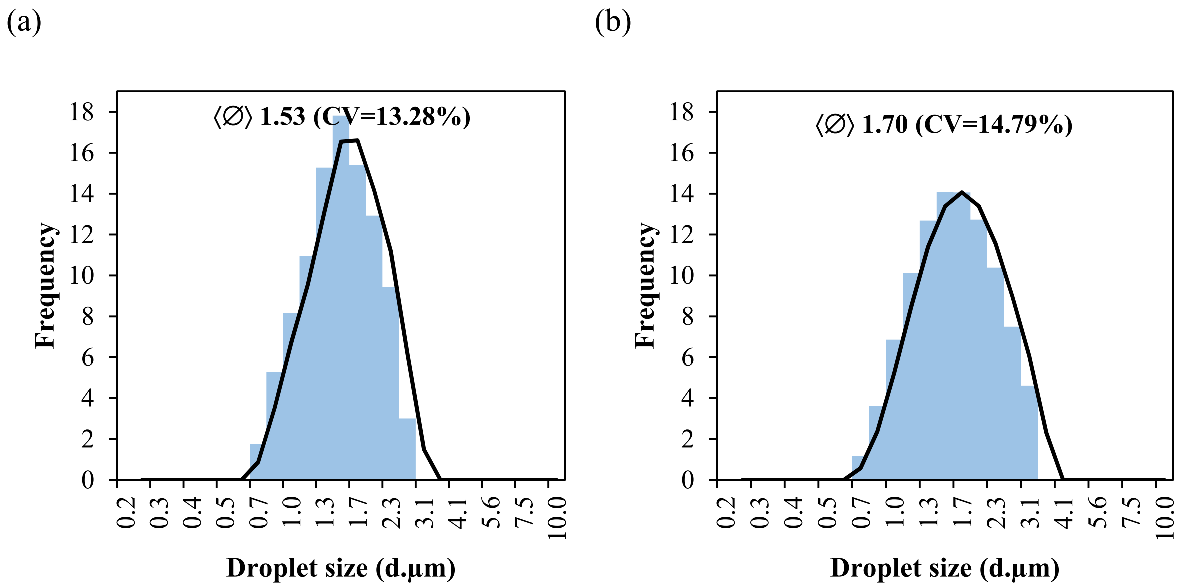

Supplementary Figure S6: Droplet size distributions with different applied bias voltages and frequencies. High voltage refers to a large amount of aqueous phase coming out from the nozzle, while the frequency slightly varies the droplet size. (a) The droplet size increases with increasing voltage from 100 V to 1,000 V at a 100-Hz frequency. (b) A higher frequency (1,000 Hz) reinforces a small droplet size at a higher voltage (1,000 V).

(a)

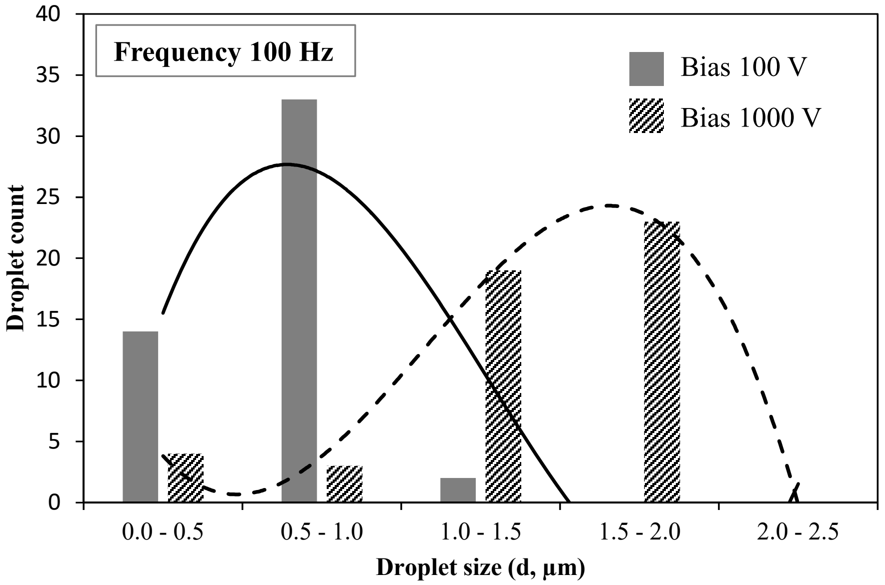

(b)

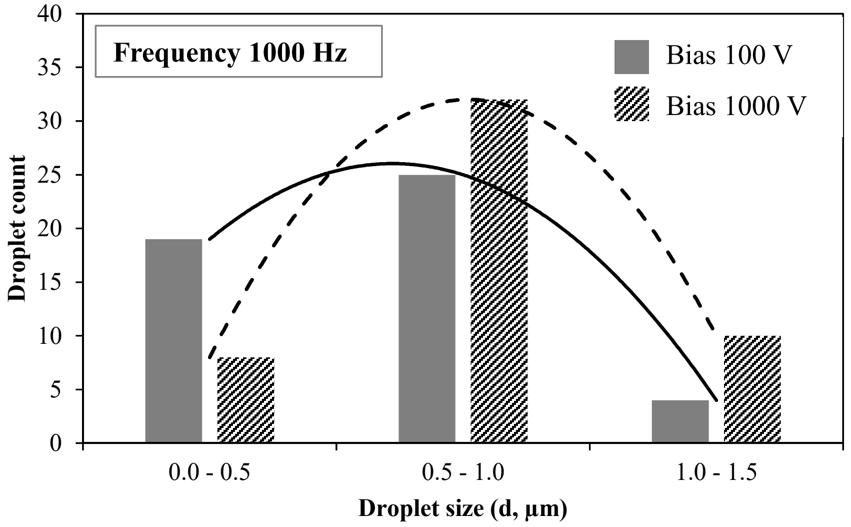

Supplementary Figure S7: Effect of voltage on DNA and GFP protein. (a) GFP was expressed in bulk using the PURE system before being electrosprayed via a glass nozzle at 50 V and 100 Hz (Off-droplet; top image). Later, GFP-encoding cDNA mixed with the PURE expression system was electrosprayed and incubated for 2 h at 37°C (On-droplet; bottom image). Confocal fluorescence microscopic images alone (left) and merged with the corresponding brightfield images (right) are shown. Scale bar: 15 µm. (b) A comparison of the time courses of GFP expression between off-droplet and on-droplet conditions.

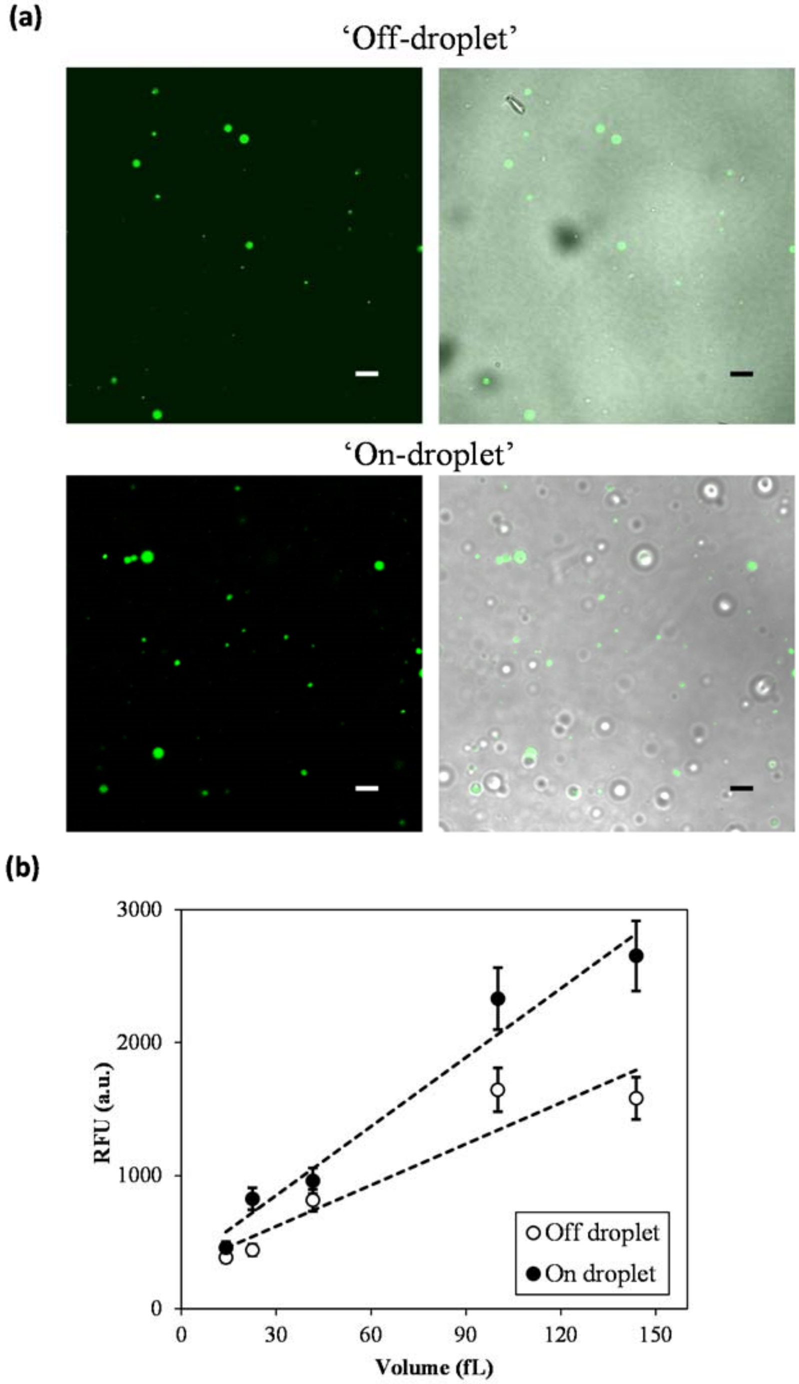

Supplementary Figure S8. *In vitro* protein expression in sub-femtoliter agarose-in-oil gel beads generated by electrospray. (a)The figure shows a confocal fluorescence image (left) and the corresponding brightfield image (right) for GFP synthesized in femtoliter agarose-in-oil gel beads. Scale bar: 10 µm. (b) Protein expression and GFP fluorescence distributions in water-in-oil droplets, agarose-in-oil droplets (w/o gelation) and agarose-in-oil gel beads (w/ gelation). [Template conc. = 35.75 nM, temperature 37ºC, droplet size = 5 µm, nozzle size 4 µm (water) and 15 µm (agarose)].

(a)

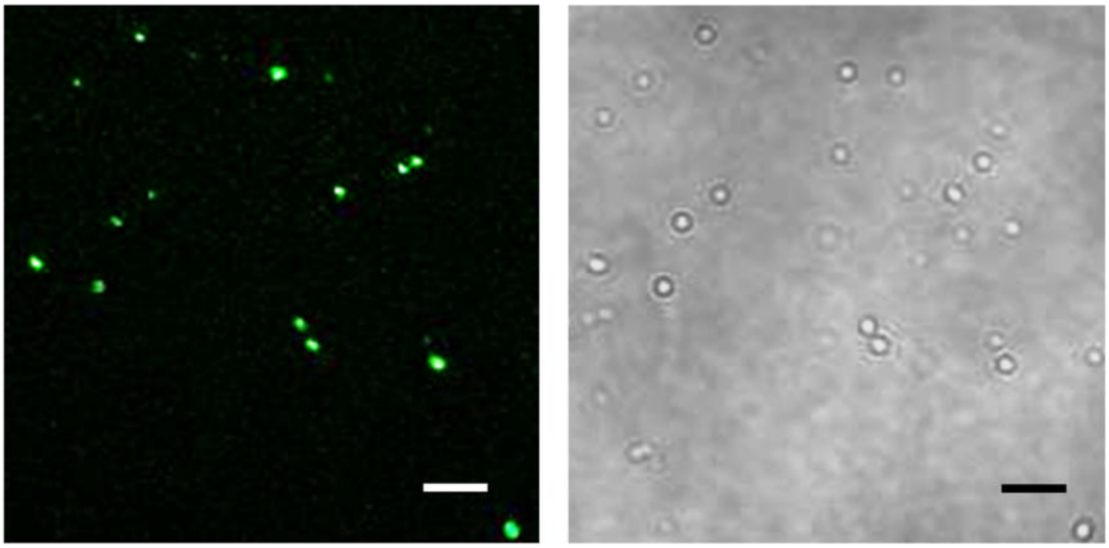

(b)

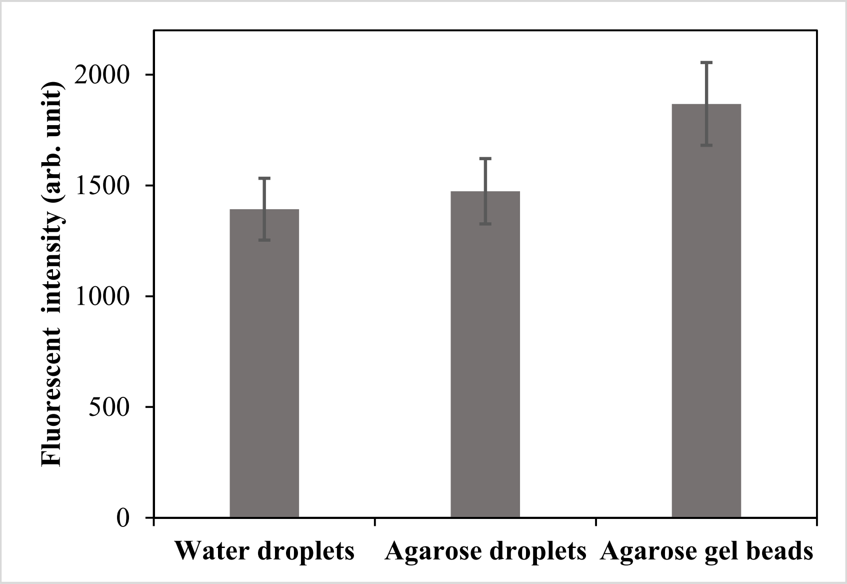

Supplementary Figure S9: 'Super-concentration' effect in femtoliter *in vitro* compartments. The extreme template dilution effect, from ~1,300 copies of the GFP encoding gene per droplet (35.75 nM) to 1 copy per 10^3 droplets (35.75 fM). The data were acquired for droplets ~5 µm in size (65 fL) or ~1.5 µm in size (2 fL).

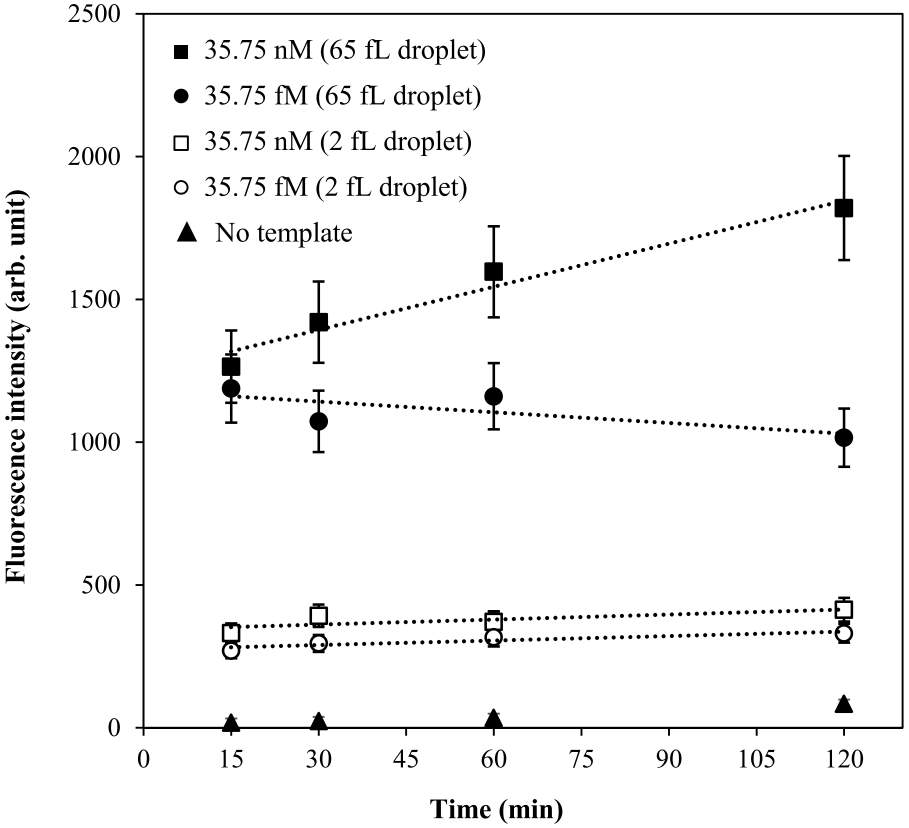

Supplementary Figure S10: Co-expression of GFP and mCherry inside water-in-oil droplets at 2 different DNA template concentrations. Fluorescence confocal microscopic images were captured using an Alexa Fluor 488 filter for GFP (left) or an Alexa Fluor 594 filter for mCherry (center); the merged images with the corresponding brightfield images are shown at the right. Encircled droplets represent the co-expression of both GFP and mCherry, which appeared as a yellow color in the merged image. Scale bar: 15 µm.

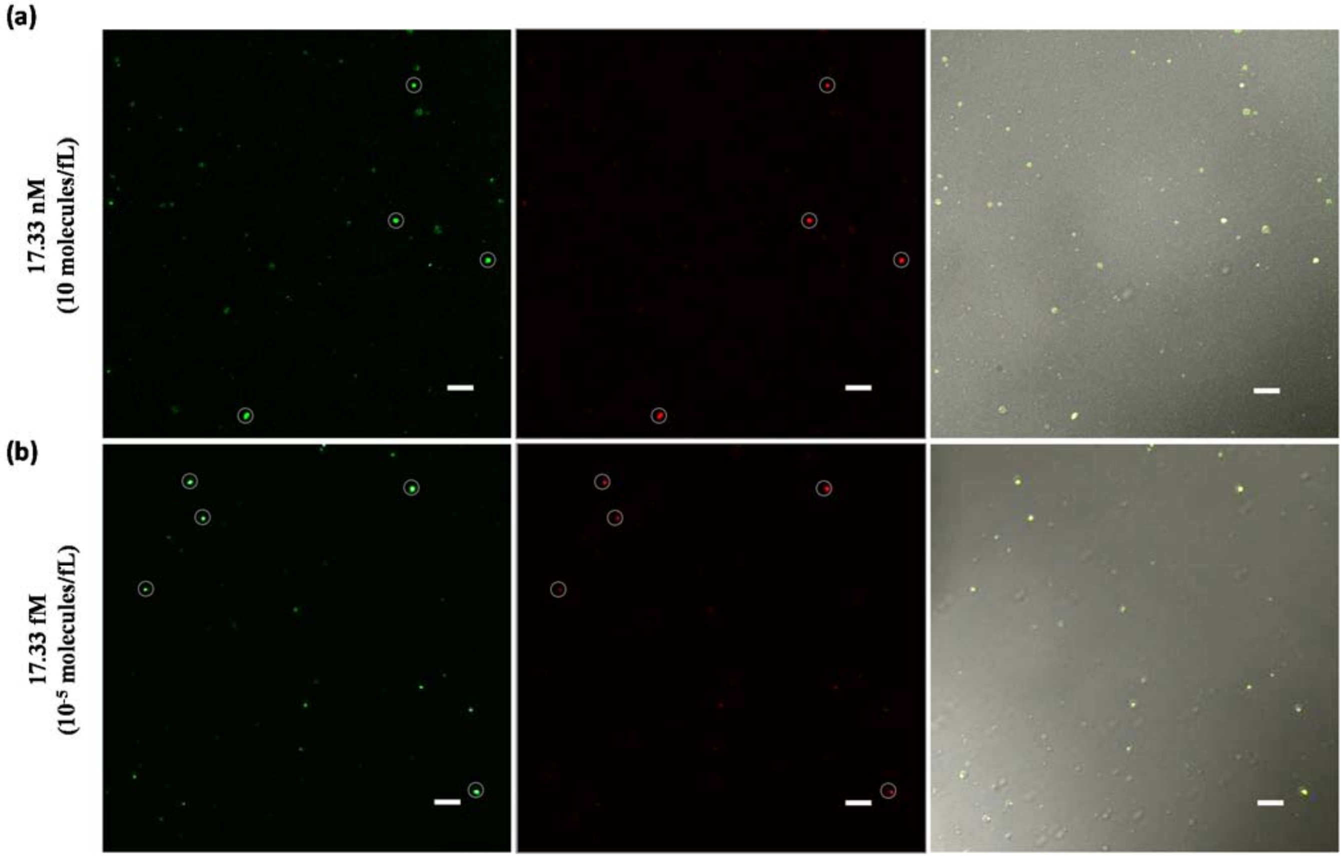

Supplementary Table S1: Comparison of three major droplet generation system.

Droplet generation method	Droplet size range	Droplet generation speed	Coefficient of variation (CV)	Technical requirement	Cost
Homogenizer	µm-to-mm	~10^9 droplets/min	>25%	Low* (Vortex)	Low
Microfluidic	>50 µm	~10^5 droplets/min	2-5%	High§ (Pump, Microfabrication)	High
Electrospray (this study)	sub-µm	10^6 to 10^8 droplets/min	<12%	Low (Nozzle, Power supply)	Moderate

*Tawfik et al, *Chem Biol* **12**, 1281–1289 (2005); §Weitz et al, *Lab Chip* **11,** 253–258 (2011)

Supplementary Table S2: Preparation of different oil viscosities* using Tegosoft DEC, ABIL EM 90 and mineral oil.

	Tegosoft DEC (Vol %)	Mineral oil (Vol %)	ABIL EM 90 (Vol %)	Viscosity (mPa.s)
1	90	7.4	2.6	8.06
2	70	22.2	7.8	12.7
3	50	37.1	12.9	23.3
4	30	51.8	18.1	39.3
5	10	66.6	23.4	73.4

**Tegosoft DEC varied from 10% to 90%, while maintaining the ABIL EM 90:mineral oil ratio at 2.85:1.*

Supplementary Table S3: Ratio of positive (with GFP expression) and negative (without GFP expression) droplets/gel beads under different conditions.

**Droplets/gel beads**	**DNA conc.**	**GFP+ve**	**GFP-ve**	**n**
Water droplets	35.75 nM	65.9%	34.1%	267
	35.75 fM	7.8%	92.2%	274
Agarose gel beads	35.75 nM	65.8%	34.2%	187

Supplementary Movie S1: Real-time video of the water-in-oil droplets produced by immersed electrospray (V_{bias} 50 V, frequency 100 Hz, nozzle size 4 µm).

Supplementary Movie S2: High-speed video of the water-in-oil droplets produced during the very first pulse by immersed electrospray. A camera at 10,000 frames per second (fps) (Phantom VR 502, Vision Research) was used to capture the images and the number of droplets was calculated using ImageJ software. The droplets produced in one pulse is found more than 55 in numbers at 50 V and nozzle size of 65 µm.

Supplementary Movie S3: High-speed video of the water-in-oil droplets generation with a fully automatic 2-dimensional motion analysis program (MotionV). A camera at 16,000 frames per second (fps) (K5, Kato Koken) was used to capture the images and the number of droplets produced per pulse is found to be 108 at 50 V using nozzle of size 4 µm.
